# Procalcitonin and C-Reactive Protein as Diagnostic Biomarkers for Bacterial Gastroenteritis: A Retrospective Analysis

**DOI:** 10.3390/jcm14072135

**Published:** 2025-03-21

**Authors:** Hyunseok Cho, Jung Ho Lee, Sung Chul Park, Sung Joon Lee, Hee-Jeong Youk, Seung-Joo Nam, Jin Myung Park, Tae Suk Kim, Ji Hyun Kim, San Ha Kim, Sang Hoon Lee

**Affiliations:** 1 Department of Pediatrics, Kangwon National University Hospital, Kangwon National University College of Medicine, Chuncheon 24289, Republic of Korea; kickboi815@hanmail.net; 2 Department of Internal Medicine, Kangwon National University Hospital, Kangwon National University College of Medicine, Chuncheon 24289, Republic of Korea; jh860726@naver.com (J.H.L.); schlp@hanmail.net (S.C.P.); joon617@kangwon.ac.kr (S.J.L.); pinetrees@daum.net (S.-J.N.); youreon.park@gmail.com (J.M.P.); greatstone@kangwon.ac.kr (T.S.K.); kimjihyun81@naver.com (J.H.K.); sing_850@naver.com (S.H.K.); 3 Department of Laboratory Medicine, Kangwon National University Hospital, Kangwon National University College of Medicine, Chuncheon 24289, Republic of Korea; jiyeonhwastar@gmail.com

**Keywords:** procalcitonin, C-reactive protein, bacterial gastroenteritis, viral gastroenteritis, diagnosis

## Abstract

**Background/Aim:** Bacterial and viral gastroenteritis present with overlapping symptoms, including vomiting, diarrhea, and abdominal pain. Stool tests have been used to differentiate between them; however, stool cultures are time-consuming and stool polymerase chain reaction (PCR) tests are expensive. The role of the clinical value of procalcitonin (PCT) and C-reactive protein (CRP) as diagnostic markers of bacterial gastroenteritis remains to be investigated. This study evaluated the diagnostic value of PCT for the early diagnosis of bacterial gastroenteritis. **Methods:** The medical records of patients diagnosed with gastroenteritis by the emergency department with positive stool PCR results confirming the diagnosis between 1 January 2020 and 31 July 2024 were retrospectively reviewed. Demographic characteristics and laboratory findings, including PCT and CRP levels, were analyzed. The area under the curve (AUC) for the diagnosis of bacterial gastroenteritis was assessed to determine the diagnostic potential of PCT and CRP. **Results:** Among the 1882 cases identified, 1435 met the inclusion criteria. CRP exhibited a sensitivity of 79.0% and specificity of 78.6% (AUC: 0.848, 95% CI: 0.815–0.881) in diagnosing bacterial gastroenteritis. In comparison, PCT showed lower sensitivity (60.3%) and specificity (62.6%) (AUC: 0.660, 95% CI: 0.614–0.706). However, in patients aged >17 years with fever (≥38 °C), PCT demonstrated an improved AUC of 0.767 (95% CI: 0.603–0.932; *p* = 0.019). **Conclusions**: CRP demonstrated moderate sensitivity in predicting bacterial gastroenteritis; however, its false-negative rate suggests limitations in clinical decision-making. While PCT may assist clinicians in identifying bacterial gastroenteritis in febrile adult patients, its diagnostic accuracy remains suboptimal, necessitating further validation through larger studies.

## 1. Introduction

Bacterial and viral gastroenteritis are characterized by overlapping symptoms such as vomiting, diarrhea, and abdominal pain [1]. However, the treatment approaches for bacterial and viral gastroenteritis differ significantly. The management of bacterial gastroenteritis involves the administration of antibiotics, whereas the management of viral gastroenteritis primarily involves providing supportive care [2]. Thus, making a prompt and accurate distinction between these conditions is crucial from the perspectives of patient care and public health concerns.

Elevations in the levels of procalcitonin (PCT), a peptide precursor to calcitonin, have been observed in patients with bacterial infections, as PCT is released in response to bacterial endotoxins and inflammatory cytokines [3,4]. However, normal or mildly elevated PCT levels are observed in viral infections due to the inhibitory effect of interferon-gamma on PCT synthesis [5]. Thus, PCT has been investigated as a biomarker to differentiate bacterial from viral infections and guide antibiotic therapy decisions [6]. Notably, studies on the diagnostic utility of PCT in patients with gastroenteritis are limited. Some studies have explored the role of PCT as a diagnostic marker for bacterial infections, with the findings revealing higher levels in patients with bacterial infections compared with those with viral infections [7,8,9,10,11,12]. During systemic inflammation, particularly in response to bacterial infections, PCT levels can rise significantly. This elevation occurs through two primary mechanisms. In the direct pathway, bacterial components such as lipopolysaccharides (LPS) directly stimulate various tissues and organs to produce PCT. In the indirect pathway, inflammatory mediators, including interleukin-6 (IL-6) and tumor necrosis factor-alpha (TNF-α), trigger PCT production. These processes lead to increased PCT synthesis in tissues beyond the thyroid, resulting in elevated serum PCT levels during bacterial infections. Conversely, viral infections do not typically induce a significant rise in PCT levels. This distinction is attributed to the production of interferon-gamma (IFN-γ) during viral infections, which inhibits PCT synthesis. PCT is a biomarker that increases in various systemic diseases, particularly in bacterial infections. Table 1 summarizes the PCT serum concentration ranges for different systemic diseases [13,14,15,16,17,18,19,20].

PCT is a potential marker of bacterial infection; however, its sensitivity and specificity vary [7,8,9,10,11,12]. Few studies have explored the utility of PCT in the differential diagnosis of bacterial gastroenteritis. Two previous studies reported conflicting results, with a sensitivity/specificity of 54.8%/52.6% and 87.03/68.75% for detecting bacterial colitis and inflammatory diarrhea, respectively [7,8]. The specificity observed in these studies was low, indicating that viral infections may slightly elevate these levels in some cases, leading to false positives. Furthermore, PCT levels may not be sufficiently elevated to distinguish viral gastroenteritis from bacterial gastroenteritis in cases with an overlap between bacterial and viral infections. Thus, PCT exhibits a promising ability to distinguish between bacterial and viral causes; however, this utility is not without limitations. Further studies are necessary to determine the role of PCT in bacterial gastroenteritis.

In this regard, this study aimed to determine whether PCT and CRP could serve as a useful biomarker for the early diagnosis of bacterial gastroenteritis in patients visiting the emergency room.

## 2. Materials and Methods

### 2.1. Study Design and Patients

The data of patients who were diagnosed with gastroenteritis by the Emergency Department of Kangwon National University Hospital and tested positive for stool PCR results between 1 January 2020 and 31 July 2024 were used in this retrospective medical chart review. Data regarding demographic characteristics, such as age, sex, and clinical symptoms (body temperature, abdominal pain, nausea, vomiting, diarrhea, and bloody stools) were analyzed. Diarrhea was defined as the passage of 3 or more loose or liquid stools per day (or more frequent passage than is normal for the individual). Other abdominal symptoms, including pain, nausea, and vomiting occurring within three days of hospitalization, were also investigated. Fever was defined as a body temperature of ≥38 °C. Only patients with stool PCR test results available within 24 h of hospitalization were included. Patients were categorized into the bacterial and viral gastroenteritis groups based on the results of real-time PCR. Furthermore, laboratory findings, including the serum PCT levels, C-reactive protein (CRP) levels, white blood cell (WBC) count, stool occult blood, and clinical symptoms, were compared between the groups. Patients with concurrent diagnoses of bacterial and viral infections and those with infections caused by ≥2 bacterial or viral pathogens were excluded to minimize confounding factors. Underlying diseases such as diabetes mellitus, thyroid disease (hyperthyroidism or hypothyroidism), hypertension, cardiovascular disease, respiratory disease, liver disease, renal disease, hyperlipidemia, and surgical history were identified through patient medical history interviews and chart reviews.

### 2.2. Laboratory Tests

A HITACHI Labospect008AS analyzer (Hitachi High-Tech Co., Tokyo, Japan) was used to measure serum CRP levels using the nephelometric method with the Qualigent CRP reagent (Sekisui Medical Co., Ltd., Tokyo, Japan). Serum PCT levels were measured using an Alinity analyzer (Abbott Diagnostics, Chicago, IL, USA) with the Alinityi B·R·A·H·M·S PCT reagent kit^®^ (Abbott, Sligo, Ireland) based on the chemiluminescence method. Stool occult blood was tested using an immunochemical fecal occult blood test (iFOBT), which specifically detects human hemoglobin in stool samples. The test was performed using the HM-JACK arc analyzer (Minaris Medical Co., Ltd., Tokyo, Japan), a fully automated fecal occult blood testing system designed for quantitative analysis.

Stool specimens were collected during the emergency department visit or after hospital admission. The test utilized EXTEL HEMO AUTO HS Latex (Minaris Medical Co., Ltd., Tokyo, Japan), a latex agglutination reagent that contains anti-human hemoglobin sheep antibodies, allowing for the specific detection of human hemoglobin. A positive result indicated the presence of occult blood in stool samples, suggesting potential gastrointestinal bleeding.

The detection of enteric pathogens was performed using the BD MAX™ Enteric Bacterial and Viral Panel (GeneOhm Science Canada ULC, Quebec City, QC, Canada). The BD MAX™ system (Becton, Dickinson and Company, Franklin Lakes, NJ, USA) can detect eight bacterial pathogens (*Campylobacter* spp., *Salmonella* spp., *Shigella* spp., enteroinvasive *Escherichia coli* [EIEC], *Plesiomonas shigelloides*, *Vibrio* spp. (*Vibrio vulnificus*, *Vibrio parahaemolyticus*, and *Vibrio cholerae*), heat-labile and heat-stable toxin-producing enterotoxigenic *Escherichia coli* [LT/ST ETEC], and *Yersinia enterocolitica*), as well as five viral pathogens (*Norovirus* genogroups I and II, *Rotavirus A*, *Adenovirus* serotypes F40/41, *Sapovirus* genogroups I, II, IV, and V, and *Human astrovirus*). Stool specimens were collected during the emergency department visit or after admission to detect bacterial and viral DNA. Stool specimens were collected during the emergency department visit or after admission to detect bacterial and viral DNA. This approach is more accurate than the standard Micro, Culture, and Sensitivity (MC&S) test that would normally be requested by conventional laboratories. The normal value range for PCT is 0~0.05 ng/mL and for CRP, it is 0~0.9 mg/dL.

### 2.3. Definition of Bacterial Gastroenteritis

The presence of symptoms of colitis and bacterial pathogens confirmed through stool PCR testing was defined as bacterial gastroenteritis [21]. The presence of viral pathogens confirmed through stool PCR testing was defined as viral gastroenteritis. Clinical manifestations of gastroenteritis include fever (body temperature of ≥38.0 °C), abdominal pain, nausea, vomiting, and diarrhea.

### 2.4. Statistical Analysis

All statistical analyses were conducted using IBM SPSS Statistics (version 25.0; IBM Corp., Armonk, NY, USA). Categorical variables are presented as numbers and percentages, whereas continuous variables are presented as the mean ± standard deviation. Categorical variables were analyzed using chi-square tests, whereas continuous variables were compared using *t*-tests. Statistical significance was set at *p* < 0.05. The diagnostic performance of PCT, CRP, WBC, segment neutrophil count, and ESR in differentiating bacterial infection from viral gastroenteritis was assessed using the receiver operating characteristic (ROC) curve analysis. Optimal cut-off values were determined using the Youden index.

### 2.5. Ethics Statement

This study was approved by the Institutional Review Board of Kangwon National University Hospital (Institutional Review Board No. KNUH-2025-01-009).

## 3. Results

### 3.1. Demographic and Clinical Characteristics

**Figure 1:**
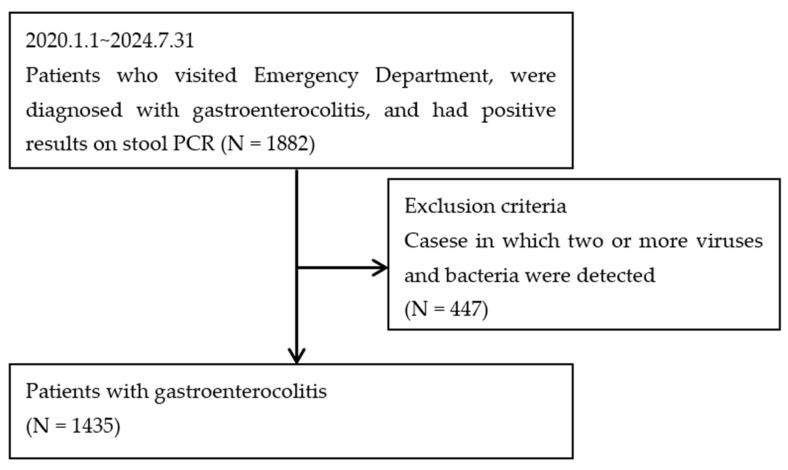
Study flow.

Among the 1882 patients with gastroenteritis that were initially identified, 1435 met the inclusion criteria (Figure 1). Among these 1435 patients, 849 (59.2%) and 586 (40.8%) were diagnosed with bacterial gastroenteritis and viral gastroenteritis, respectively. The mean age of the patients diagnosed with bacterial gastroenteritis was significantly higher (mean age: 35.71 ± 29.10 years) than that of those diagnosed with viral gastroenteritis (mean age: 13.13 ± 23.87 years; *p* < 0.001). The proportion of male patients in the bacterial gastroenteritis group (55.7%) was higher than that observed in the viral gastroenteritis group (51.5%; *p* < 0.001). Clinical symptoms such as fever (62.4% vs. 31.6%, *p* < 0.001), abdominal pain (90.5% vs. 73.4%, *p* < 0.001), diarrhea (88.1% vs. 61.1%, *p* < 0.001), and stool occult blood, detected using the guaiac fecal occult blood test (gFOBT) (7.6% vs. 2.9%, *p* < 0.001), were observed significantly more commonly among patients with bacterial gastroenteritis. In contrast, nausea (55.7% vs. 80.7%, *p* < 0.001) and vomiting (35.2% vs. 78.9%, *p* < 0.001) were observed more commonly in patients with viral gastroenteritis.

The laboratory parameters of patients with bacterial gastroenteritis were higher than those of patients with viral gastroenteritis: neutrophil count (74.52 ± 14.27% vs. 63.32 ± 21.99%, *p* < 0.001), CRP levels (8.28 ± 7.24 mg/dL vs. 1.68 ± 3.57 mg/dL, *p* < 0.001), and PCT levels (1.72 ± 10.61 ng/mL vs. 0.34 ± 0.96 ng/mL, *p* < 0.001) (Table 2).

The prevalence of underlying diseases such as diabetes mellitus (9.3% vs. 3.6%, *p* < 0.001), hypertension (16.7% vs. 5.5%, *p* < 0.001), cerebrovascular disease (2.9% vs. 1.2%, *p* < 0.001), renal disease (2.0% vs. 0.5%, *p* = 0.018), and hyperlipidemia (9.4% vs. 2.4%, *p* < 0.001) was also higher in the bacterial gastroenteritis group.

### 3.2. Pathogen Detection

Table 2 and Table 3 present the causative pathogens identified through PCR testing. *Campylobacter* spp. was the most common pathogen (*n* = 490, 57.7%) in the bacterial gastroenteritis group, followed by *Salmonella* spp. (*n* = 165, 19.4%), *Clostridioides difficilet* oxin A/B (*n* = 80, 9.4%), STEC (Shiga-like toxin-producing *Escherichia coli*) stx1/stx2 (*n* = 35, 4.1%), *Yersinia enterocolitica* (*n* = 23, 2.7%), EPEC (enteropathogenic *E. coli*) (*n* = 16, 1.9%), Enterotoxigenic *E. coli* (ETEC; *n* = 11, 1.3%), *Vibrio* spp. (*n* = 11, 1.3%), *Plesiomonas shigelloides* (*n* = 10, 1.2%), enteroaggregative *E. coli* (EAEC; *n* = 5, 0.6%), and *Shigella* spp. (*n* = 3, 0.4%) (Table 3).

Norovirus GI/GII was the most common pathogen (*n* = 325, 55.5%) in the viral gastroenteritis group, followed by rotavirus (*n* = 86, 14.7%), adenovirus 40/41 (*n* = 61, 10.4%), sapovirus (*n* = 61, 10.4%), and astrovirus (*n* = 53, 9.0%) (Table 4).

### 3.3. ROC Analysis

**Figure 2:**
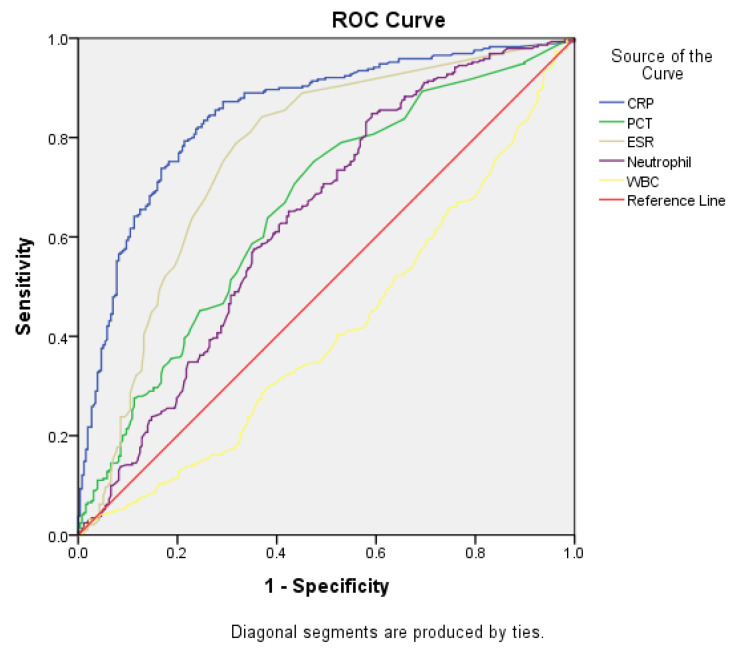
Receiver operating characteristic curve of CRP, PCT, ESR, neutrophil count, and WBC for differentiating between bacterial and viral gastroenteritis. CRP, C-reactive protein; PCT, procalcitonin; ESR, erythrocyte sedimentation rate; WBC, white blood cells.

Table 5 and Figure 2 summarize the findings of the ROC curve analysis for CRP, PCT, ESR, neutrophil count, and WBC count in differentiating bacterial infections from viral gastroenteritis. The area under the curve (AUC) for CRP was 0.848 (95% confidence interval [CI], 0.815–0.881), with a sensitivity and specificity of 79.0% and 78.6%, respectively, at a cut-off value of 1.8 mg/dL. The AUC for PCT for diagnosing bacterial gastroenteritis was 0.660 (95% CI, 0.614–0.706), with a sensitivity and specificity of 60.3% and 62.6%, respectively, at a cut-off value of 0.1 ng/mL. The AUC for ESR was 0.763 (95% CI, 0.721–0.805), with a sensitivity and specificity of 71.0% and 72.8%, respectively, at a cut-off value of 10.5 mg/dL. The AUC for the neutrophil count was 0.638 (95% CI, 0.591–0.684), with a sensitivity and specificity of 60.7% and 67.0%, respectively, at a cut-off value of 74.4%.

ROC analysis revealed variations in the AUC values depending on symptoms and age groups (Table 6 and Supplementary Figure S1). Specifically, the AUC for PCT improved to 0.767 (95% CI, 0.603–0.932) in patients aged >17 years with fever (body temperature ≥ 38 °C; total *n* = 697, febrile *n* = 332). The sensitivity and specificity of PCT were 68.8% and 71.4%, respectively, at a cut-off value of 0.1 ng/mL. Other inflammatory markers, including the CRP level, ESR, neutrophil count, and WBC, were not statistically significant in this subgroup.

## 4. Discussion

In the present study, CRP demonstrated higher overall sensitivity and specificity in diagnosing bacterial gastroenteritis. PCT, while less sensitive overall, may have a role in specific populations, particularly febrile adults (>17 years, fever ≥ 38 °C). These findings highlight the complementary roles of CRP and PCT in clinical decision-making.

The potential of PCT as a biomarker in the clinical diagnosis of infections, particularly bacterial infections, has garnered an increasing amount of attention [3]. PCT is a precursor of calcitonin, which is produced by thyroid C-cells [22]. PCT levels increase significantly in response to bacterial infections; thus, they are a valuable marker for distinguishing bacterial infections from other inflammatory processes, such as viral infections or non-infectious conditions [23].

The differentiation of bacterial gastroenteritis caused by pathogens such as *Salmonella*, *Shigella*, *Campylobacter*, *E. coli*, and *Clostridioides difficile* from viral gastroenteritis and other non-infectious gastrointestinal conditions can be challenging [24]. Unfortunately, conventionally used diagnostic methods, such as stool culture, PCR, and serology, are time-consuming and expensive, which can delay appropriate treatment. Furthermore, they exhibit limited sensitivity. CRP is an inflammatory marker synthesized in the liver in response to elevated cytokine levels. Its production begins 4–6 h after the onset of inflammation or tissue damage, with values doubling approximately every 8 h. The properties of PCT are similar to those of CRP, i.e., it increases earlier, is more specific, and its levels are correlated with the severity of infection [25].

PCT is predominantly produced within thyroid C-cells and is converted to calcitonin before entering the systemic circulation in healthy individuals. Consequently, very low serum PCT levels (<0.02 ng/mL) are observed in healthy individuals [5]. In contrast, PCT is produced through an “alternative pathway” in non-thyroid tissues such as the spleen, kidneys, adipocytes, pancreas, colon, brain, and lungs in patients with systemic bacterial infections. These parenchymal tissues lack the processing pathway required to convert PCT to calcitonin, thereby allowing PCT to enter the systemic circulation and elevate the serum PCT levels. Serum PCT levels are typically <0.1 ng/mL in healthy individuals. Thus, PCT levels of >0.25 ng/mL may indicate the presence of a bacterial infection [5]. PCT levels of >0.5 ng/mL are generally suggestive of a bacterial infection, whereas levels of >2 ng/mL are often associated with more severe bacterial infections [26]. PCT levels are correlated with the severity of bacterial infections [4]. Elevated PCT levels may indicate more severe or complicated conditions such as sepsis or severe colitis in patients with bacterial gastroenteritis [27]. A meta-analysis targeting acute pancreatitis revealed that the sensitivity and specificity of PCT were lower than those of other biomarkers such as CRP or fecal calprotectin; however, it serves as a helpful adjunct in certain clinical scenarios to make appropriate treatment decisions [28]. Monitoring PCT levels over time can provide valuable insights into the progression of the infection and response to treatment [29]. Thus, PCT levels can guide antibiotic therapy by distinguishing bacterial infections from gastrointestinal symptoms caused by non-bacterial factors [6]. Elevated PCT levels may support the decision to initiate antibiotic treatment in cases wherein bacterial infection is suspected. However, low PCT levels in patients with suspected bacterial infections can help avoid unnecessary antibiotic use, which will aid in reducing the emergence of antibiotic-resistant bacteria.

The utility of PCT as a potential biomarker for differentiating bacterial gastroenteritis from viral gastroenteritis was evaluated in this context. A minimal increase in PCT levels is observed in patients with viral infections, such as those caused by norovirus or rotavirus. In contrast, significant elevation is observed in patients with bacterial infections. Although PCT levels are generally higher in patients with bacterial gastroenteritis compared with that in patients with viral gastroenteritis, they can vary depending on the severity of infection and the specific pathogen involved. Thus, the sensitivity and specificity of PCT for detecting bacterial infections, including gastroenteritis, varies, leading to false-positive results in some cases [7,8,9,10,11,12]. Furthermore, the elevated PCT levels observed in patients with non-infectious conditions such as inflammatory bowel disease [27], pancreatitis [30,31], or postsurgical conditions [32,33] may potentially complicate interpretation in certain patient populations. The response of PCT to bacterial infections may be blunted in immunocompromised patients [34,35]; thus, it also increases the likelihood of false negatives. PCT is particularly useful for ruling out bacterial infections at low levels. PCT levels combined with clinical findings can help guide decisions regarding the initiation or avoidance of antibiotics in cases wherein the etiology of gastroenteritis is unclear [36]. Expert and systematic reviews recommend using PCT as part of a comprehensive diagnostic workup for infections, such as severe cases of gastroenteritis, to minimize the unnecessary use of antibiotics for viral infections [37,38]. Shin et al. [8] reported that PCT was a better diagnostic biomarker for inflammatory diarrhea (odds ratio, 1.321; AUC, 0.797) than CRP (odds ratio, 1.145; AUC, 0.697). In contrast, Lee et al. [7] reported that PCT and CRP levels could not be used to distinguish between bacterial and non-bacterial colitis. However, both of these studies were retrospective studies conducted at single institutions; thus, overall evidence remains mixed. Further large-scale prospective studies must be conducted in the future to establish the precise role of PCT in the diagnosis of bacterial gastroenteritis.

The present study retrospectively evaluated the diagnostic value of PCT and CRP in differentiating bacterial gastroenteritis from viral gastroenteritis in patients who visited the emergency department. CRP demonstrated higher overall sensitivity and specificity in diagnosing bacterial gastroenteritis. PCT, while less sensitive overall, may have a role in specific populations, particularly febrile adults (>17 years, fever ≥ 38 °C). In other words, CRP demonstrated a sensitivity of 79.0% and specificity of 78.6% (AUC: 0.848, 95% CI, 0.815–0.881) in diagnosing bacterial gastroenteritis, leading to a false-negative rate of 21%. In comparison, PCT exhibited lower sensitivity (60.3%) and specificity (62.6%), resulting in a false-negative rate approaching 40% (AUC: 0.660, 95% CI, 0.614–0.706). In patients aged > 17 years with fever (≥38 °C), PCT demonstrated an improved AUC of 0.767 (95% CI: 0.603–0.932; *p* = 0.019), suggesting potential utility in this subgroup. These findings highlight the complementary roles of CRP and PCT in clinical decision-making. *Campylobacter* spp. (57.7%) and *Salmonella* spp. (19.4%) were the primary bacterial pathogens identified, whereas norovirus (55.5%) was the most common viral pathogen. In comparison to viral gastroenteritis, which is often associated with mild or no fever, bacterial gastroenteritis frequently presents with high fever [39]. In contrast to previous studies that focused on sepsis or generalized bacterial infections, the present study specifically evaluated its role in gastroenteritis. Notably, the present study included a relatively large patient population of various age groups, including children, in contrast to previous studies targeting bacterial colitis or inflammatory diarrhea. The findings of this study indicate that both CRP and PCT have limited clinical utility in diagnosing bacterial gastroenteritis. Given these limitations, PCT is not a clinically useful biomarker for bacterial gastroenteritis. The high false-negative rate implies that even if PCT is low, clinicians are unlikely to rule out bacterial gastroenteritis and withhold antibiotics, particularly in febrile patients. As a result, PCT testing does not provide actionable clinical guidance in this setting.

PCT is primarily a marker for bacterial infections; however, it can also be elevated in patients with certain non-infectious inflammatory conditions, such as trauma, surgery, burns, cancer, autoimmune diseases, chronic kidney disease, heart failure, and myocardial infarction, or following the administration of medications such as immunosuppressants and chemotherapy agents [40]. Thus, interpreting PCT levels may be complicated in some cases. Several underlying diseases, such as diabetes, hypertension, cerebrovascular disease, and renal disease, were more prevalent in the bacterial gastroenteritis group than in the viral gastroenteritis group in the present study. The effect of these diseases on PCT levels cannot be ruled out. All bacterial infections do not result in the same degree of PCT elevation. For instance, infections caused by *Clostridioides difficile* may not result in a significant increase in PCT compared with that caused by other enteric bacteria, making its diagnostic utility dependent on the specific pathogen [41]. Moreover, PCT is not exclusive to gastrointestinal infections and can be elevated in other bacterial infections, such as respiratory tract infections or sepsis, as well as in severe systemic inflammatory responses [40]. Thus, it cannot be used as a standalone diagnostic tool; rather, it can be used in conjunction with clinical findings and other laboratory tests. Future research should focus on identifying more reliable biomarkers or integrating CRP and PCT with other diagnostic approaches to improve sensitivity and specificity. Until then, these markers should be used cautiously and not as standalone tests in clinical practice.

This study has some limitations. First, the single-center, retrospective design may have limited the generalizability of the findings. Second, PCT levels can be elevated in patients with comorbid conditions. However, there were limitations in fully analyzing the underlying conditions or medications as this was a retrospective study. Third, the sensitivity of stool PCR testing is generally high; however, some pathogens may not have been detected. Prospective multicenter studies must be conducted in the future to confirm these findings and establish standardized PCT thresholds tailored to diverse patient populations.

## 5. Conclusions

In conclusion, CRP showed a moderate diagnostic value for bacterial gastroenteritis; however, its sensitivity remains insufficient for definitive diagnosis. In febrile adult patients with suspected bacterial gastroenteritis, PCT may provide additional diagnostic insights, but its high false-negative rate necessitates cautious interpretation. Further large-scale prospective studies are needed to refine its clinical utility.

## Figures and Tables

**Table 1:** PCT serum concentration ranges for different systemic diseases.

Disease	PCT Serum Level Range (ng/mL)	Author
Normal	<0.05	Samsudin I et al. [13]
Bacterial Sepsis	>2.0	Endo S et al. [14]
Community-Acquired Pneumonia	0.1–0.5	Schuetz P et al. [15]
Severe Systemic Inflammation	2.0–10.0	Sinha M et al. [16]
Viral Infections	<0.5	Azijli K et al. [17]
Postoperative Fever	0.1–0.5	Hunziker S et al. [18]
Pancreatitis	0.25–0.5	Gurda-Duda A et al. [19]
Urinary Tract Infections	0.25	Van Nieuwkoop C et al. [20]

**Table 2:** Patient characteristics in the bacterial and viral gastroenteritis groups.

Characteristics	Bacterial Gastroenteritis	Viral Gastroenteritis	*p*-Value
	(*n* = 849)	(*n* = 586)	
Age (year, mean ± SD)	35.71 ± 29.10	13.13 ± 23.87	<0.001
Male sex (*n*, %)	473 (55.7%)	302 (51.5%)	<0.001
Symptom			
Fever (BT ≥ 38 °C)	492 (62.4%)	174 (31.6%)	<0.001
Abdominal pain (*n*, %)	582 (90.5%)	179 (73.4%)	<0.001
Nausea (*n*, %)	352 (55.7%)	409 (80.7%)	<0.001
Vomiting (*n*, %)	221 (35.2%)	403 (78.9%)	<0.001
Diarrhea (*n*, %)	712 (88.1%)	319 (61.1%)	<0.001
Stool occult blood (*n*, %)	62 (7.6%)	15 (2.9%)	<0.001
Laboratory finding			
WBC count (×10^3^/μL)	10,421.77 ± 4977.77	11,900.05 ± 5679.06	<0.001
Neutrophil count (%)	74.52 ± 14.27	63.32 ± 21.99	<0.001
CRP (mg/dL)	8.28 ± 7.24	1.68 ± 3.57	<0.001
ESR (mm/h)	22.34 ± 16.80	11.43 ± 16.36	<0.001
Procalcitonin (ng/mL)	1.72 ± 10.61	0.34 ± 0.96	<0.001
Underlying disease			
DM	79 (9.3%)	21 (3.6%)	<0.001
Thyroid disease	11 (1.3%)	4 (0.7%)	0.262
HTN	142 (16.7%)	32 (5.5%)	<0.001
CVD	25 (2.9%)	7 (1.2%)	0.027
Respiratory disease	7 (0.8%)	3 (0.5%)	0.484
Liver disease	4 (0.5%)	1 (0.2%)	0.342
Renal disease	17 (2.0%)	3 (0.5%)	0.018
Hyperlipidemia	80 (9.4%)	14 (2.4%)	<0.001
Surgical history	24 (2.8%)	9 (1.5%)	0.273

BT, body temperature; WBC, white blood cell; CRP, C-reactive protein; ESR, erythrocyte sedimentation rate; DM, diabetes mellitus; HTN, hypertension; CVD, cerebrovascular disease.

**Table 3:** Distribution of bacteria detected through stool polymerase chain reaction testing.

Type	Data
*Campylobacter* spp.	490 (57.7%)
*Clostridioides difficile* toxin A/B	80 (9.4%)
EAEC (enteroaggregative *E. coli*)	5 (0.6%)
EPEC (enteropathogenic *E. coli*)	16 (1.9%)
ETEC (enterotoxigenic *E. coli*)	11 (1.3%)
*Plesiomonasshigelloides*	10 (1.2%)
*Salmonella* spp.	165 (19.4%)
*Shigella* spp.	3 (0.4%)
STEC (Shiga-like toxin-producing *E. coli*) stx1/stx2	35 (4.1%)
*Vibrio* spp.	11 (1.3%)
*Yersinia enterocolitica*	23 (2.7%)
Total	849 (100.0%)

**Table 4:** Distribution of viruses detected through stool polymerase chain reaction testing.

Type	Data
Adenovirus 40/41	61 (10.4%)
Astrovirus	53 (9.0%)
Norovirus GI/GII	325 (55.5%)
Rotavirus	86 (14.7%)
Sapovirus	61 (10.4%)
Total	586 (100.0%)

Values are presented as number (%).

**Table 5:** Receiver operating characteristic analysis of CRP, PCT, ESR, neutrophil count, and WBC for predicting bacterial gastroenteritis.

Index	AUC (95% CI)	Cut-Off	Sensitivity (%)	Specificity (%)	*p*-Value
CRP	0.848 (0.815–0.881)	1.8 (mg/dl)	79.0%	78.6%	<0.0001
PCT	0.660 (0.614–0.706)	0.1 (ng/mL)	60.3%	62.6%	<0.0001
ESR	0.763 (0.721–0.805)	10.5 (mm/h)	71.0%	72.8%	<0.0001
Neutrophil count	0.638 (0.591–0.684)	74.4 (%)	60.7%	67.0%	<0.0001
WBC	0.412 (0.364–0.460)	10.75 (×10^3^/μL)	41.7%	42.4%	<0.0001

CRP, C-reactive protein; PCT, procalcitonin; ESR, erythrocyte sedimentation rate; WBC, white blood cell; AUC, area under the curve; CI, confidence interval.

**Table 6:** Receiver operating characteristic analysis of CRP, PCT, ESR, neutrophil count, and WBC for predicting bacterial gastroenteritis among patients aged > 17 years old with fever (BT ≥ 38 °C).

Index	AUC (95% CI)	Cut-Off	Sensitivity (%)	Specificity (%)	*p*-Value
CRP	0.715 (0.479–0.951)	8.98	63.4%	71.4%	0.059
PCT	0.767 (0.603–0.932)	0.1	68.8%	71.4%	0.019
ESR	0.683 (0.396–0.970)	13.5	71.0%	71.4%	0.108
Neutrophil count	0.445 (0.178–0.713)	84.4	41.9%	42.9%	0.632
WBC	0.510 (0.269–0.751)	10,500	41.9%	42.9%	0.930

CRP, C-reactive protein; PCT, procalcitonin; ESR, erythrocyte sedimentation rate; WBC, white blood cell; BT, body temperature; AUC, area under the curve; CI, confidence interval.

## Data Availability

Data are available upon request.

## Supplementary Materials

The following supporting information can be downloaded at https://www.mdpi.com/article/10.3390/jcm14072135/s1, Figure S1: Receiver operating characteristic curve of CRP, PCT, ESR, neutrophil count, and WBC for differentiating between bacterial and viral gastroenteritis among patients aged >17 years old with fever (BT ≥ 38 °C); Table S1: Receiver operating characteristic analysis of CRP, PCT, ESR, neutrophil count, and WBC to differentiate between bacterial and viral gastroenteritis according to the symptoms.
