# Supplementary material for: Procalcitonin and C-Reactive Protein as Diagnostic Biomarkers for Bacterial Gastroenteritis: A Retrospective Analysis
Source: J Clin Med. 2025 Mar 21;14(7):2135. doi: 10.3390/jcm14072135 (PMC11989930; doi:10.3390/jcm14072135)
Supplement: Supplementary file 1 [file jcm-14-02135-s001.zip › jcm-3505824-supplementary.pdf]

Journal of Clinical Medicine

MDPI

Supplementary Table S1. Receiver operating characteristic analysis of CRP, PCT, ESR, neutrophil count, and WBC to differentiate between bacterial and viral gastroenteritis according to the symptoms (fever, diarrhea, vomiting, nausea, and abdominal pain).

Age	Symptom	Area Under the Curve				
		WBC	Neutrophil Count	CRP	ESR	PCT
All ages		0.412 (0.364–0.460) *	0.638 (0.591–0.684) *	0.848 (0.815–0.881) *	0.763 (0.721–0.805) *	0.660 (0.614–0.706) *
<17		0.455 (0.395–0.516)	0.595 (0.537–0.653) *	0.817 (0.772–0.862) *	0.741 (0.689–0.793) *	0.676 (0.620–0.732) *
≥17		0.472 (0.360–0.585)	0.474 (0.348–0.599)	0.758 (0.661–0.856) *	0.594 (0.451–0.737)	0.679 (0.661–0.856) *
All ages	BT ≧ 38 °C	0.444 (0.369–0.519)	0.641 (0.564–0.717) *	0.814 (0.755–0.872) *	0.690 (0.614–0.766) *	0.631 (0.557–0.706) *
<17	BT ≧ 38 °C	0.635 (0.549–0.721)	0.576 (0.488–0.665)	0.773 (0.700–0.846) *	0.655 (0.568–0.742) *	0.635 (0.549–0.721) *
≥17	BT ≧ 38 °C	0.510 (0.269–0.751)	0.445 (0.178–0.713)	0.715 (0.479–0.951)	0.683 (0.396–0.970)	0.767 (0.603–0.932) *
All ages	Diarrhea	0.423 (0.363–0.483) *	0.669 (0.608–0.731) *	0.885 (0.851–0.919) *	0.802 (0.753–0.850) *	0.670 (0.613–0.727) *
<17	Diarrhea	0.434 (0.356–0.512)	0.661 (0.587–0.735) *	0.864 (0.814–0.915) *	0.795 (0.734–0.856) *	0.681 (0.608–0.754) *
≥17	Diarrhea	0.510 (0.390–0.630)	0.448 (0.314–0.583)	0.830 (0.749–0.911) *	0.655 (0.503–0.806) *	0.733 (0.636–0.831) *
All ages	Vomiting	0.465 (0.391–0.538)	0.660 (0.592–0.728) *	0.899 (0.857–0.940) *	0.816 (0.761–0.872) *	0.732 (0.668–0.797) *
<17	Vomiting	0.474 (0.380–0.569) *	0.602 (0.510–0.693) *	0.867 (0.812–0.922) *	0.790 (0.7130–0.868) *	0.725 (0.641–0.810) *
≥17	Vomiting	0.671 (0.477–0.865)	0.484 (0.262–0.707)	0.906 (0.814–0.998) *	0.725 (0.483–0.967)	0.822 (0.667–0.947) *
All ages	Nausea	0.430 (0.366–0.494) *	0.661 (0.602–0.720) *	0.909 (0.876–0.943) *	0.816 (0.768–0.864) *	0.698 (0.641–0.756) *
<17	Nausea	0.458 (0.374–0.541)	0.623 (0.545–0.700) *	0.886 (0.842–0.930) *	0.791 (0.726–0.857) *	0.715 (0.643–0.788) *
≥17	Nausea	0.615 (0.459–0.770)	0.434 (0.242–0.627)	0.891 (0.804–0.977) *	0.698 (0.485–0.911) *	0.789 (0.667–0.910) *
All ages	Abdominal pain	0.416 (0.341–0.490) *	0.483 (0.404–0.563)	0.840 (0.787–0.893) *	0.761(0.692–0.830) *	0.640 (0.568–0.713) *
<17	Abdominal pain	0.414 (0.319–0.508)	0.461 (0.363–0.560)	0.845 (0.779–0.910) *	0.782(0.702–0.862) *	0.642 (0.548–0.735) *
≥17	Abdominal pain	0.504 (0.374–0.634)	0.445 (0.306–0.584)	0.768 (0.651–0.885) *	0.614(0.445–0.783)	0.707 (0.598–0.815) *

* p-value < 0.5. CRP, C-reactive protein; PCT, procalcitonin; ESR, erythrocyte sedimentation rate; WBC, white blood cell.

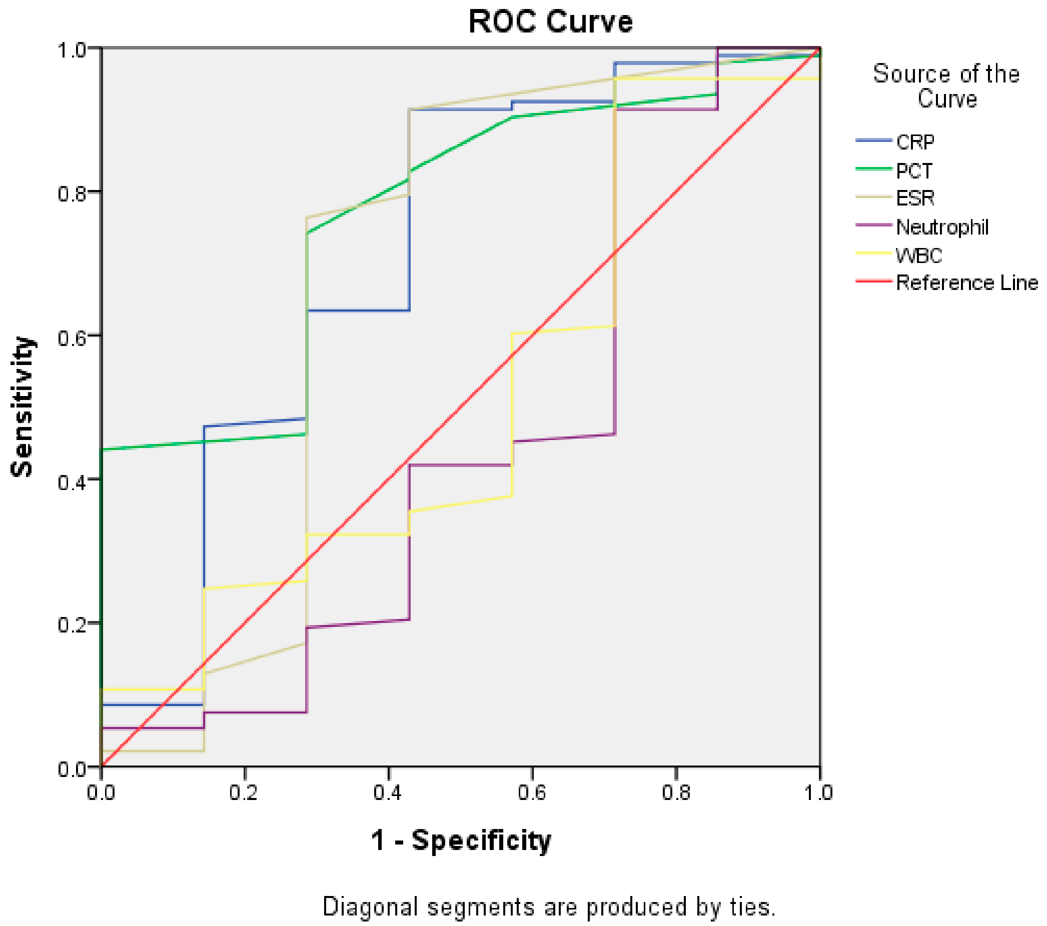

Supplementary Figure S1. Receiver operating characteristic curve of CRP, PCT, ESR, neutrophil count, and WBC for differentiating between bacterial and viral gastroenteritis among patients aged >17 years old with fever (BT ≧ 38 °C). CRP, C-reactive protein; PCT, procalcitonin; ESR, erythrocyte sedimentation rate; WBC, white blood cells.
